# Pulmonary Arterial Hypertension and Insulin Resistance

**DOI:** 10.4172/1747-0862.S1-015

**Published:** 2014-01-23

**Authors:** Elisa A Bradley, David Bradley

**Affiliations:** 1 Division of Cardiovascular Medicine, The Ohio State University Wexner Medical Center and Nationwide Children’s Hospital, Columbus, OH, USA; 2 Division of Endocrinology, Diabetes and Metabolism, The Ohio State University Wexner Medical Center, Columbus, OH, USA

**Keywords:** Insulin resistance, Pulmonary arterial hypertension

## Abstract

The clinical recognition of pulmonary arterial hypertension (PAH) is increasing, and with recent therapeutic advances, short-term survival has improved. In spite of these advances, however, PAH remains a disease with substantial morbidity and long-term mortality. The pathogenesis of PAH involves a complex interaction of local and distant cytokines, growth factors, co-factors, and transcription factors occurring in the right genetic and environmental setting. These factors ultimately lead to the detrimental changes in vascular anatomy and function seen in PAH patients. An important association between obesity/insulin resistance and PAH has recently been identified. Both conditions occur in the presence of a chronic low-grade inflammatory state, and although it is unlikely that a single pathway is solely responsible for the observed association, deficiencies in adiponectin, apolipoprotein E (ApoE) and peroxisome proliferator-activator receptor gamma (PPAR-γ) activity likely play a prominent role. Although incompletely understood, it is clear that further investigation is warranted and the role of weight loss and improved glycemic control in the treatment of at-risk patients with PAH and obesity should be determined.

## Introduction

Pulmonary arterial hypertension (PAH) is a disease characterized by abnormal pulmonary vascular remodeling, endothelial vasoconstriction, and thrombosis in-situ, ultimately leading to elevated pulmonary vascular resistance (PVR) [1–3]. These adverse changes to the vasculature affect right ventricular function and can progress to cor pulmonale, or right heart failure. Even with new PAH-specific medical therapy (i.e. phosphodiesterase inhibitors, endothelin receptor antagonists, and prostacyclins), this remains a disease with significant morbidity and mortality [4]. An important distinction must be made between PAH and other causes of pulmonary hypertension (PH), which can be divided into five subtypes by the World Health Organization (WHO) Dana Point classification system (Table 1) [5]. The more general term PH reflects the underlying presence of high pulmonary vascular pressure from any source, but is most commonly seen, in a clinical setting, with left sided heart disease secondary to systolic heart failure, diastolic dysfunction, and/or left sided valve disease (WHO group 2). This is often referred to as pulmonary venous hypertension or PVH. PAH, in contrast, is a distinct subtype of PH (WHO group 1) leading to elevated resistance in the pulmonary vascular bed, and is defined by the following criteria: mean pulmonary artery (mPA) pressure>25 mmHg at rest in the setting of normal pulmonary capillary wedge pressure (<15 mmHg) with pulmonary vascular resistance (PVR)>3 Wood units [6].

Causes of PAH include: idiopathic (without identifiable risk factors), heritable, and drug/toxin-induced, among others (Table 1). PAH can also occur in the background of a preexisting medical condition such as connective tissue disease, infectious disease (Human Immunodeficiency Virus, Schistosomiasis, etc.), cirrhosis, or congenital heart disease. Identification of a single mechanistic process is particularly challenging in the setting of multiple etiologies and the complex interplay between cellular, environmental, and genetic factors that contribute to the disease process. Symptoms, including shortness of breath and decreased exercise tolerance, often develop gradually, leading to a delay in diagnosis and treatment. In fact, some patients initially present only after a syncopal episode, reflecting the presence of advanced disease with dramatic increases in PA pressure and low cardiac output (CO).

Recent diagnostic and technological advances have offered some insight into the underlying pathogenesis of this disease, providing the potential for novel therapeutic targets. One of the most intriguing aspects of research is the apparent association between PAH and insulin resistance (IR) [7,8]. In the following review, we aim to summarize the existing data and highlight some potential pathophysiologic mechanisms of PAH and its association with obesity and obesity-related IR.

## PAH: Epidemiology and Pathophysiology

PAH, as defined by the above criteria, is a relatively rare disorder. Although uncommon, it is increasingly being recognized and diagnosed. Prevalence data from a recent French registry study suggest that 15 persons per million are affected by the disease [9]. Most of the available epidemiologic and outcome data from the United States (US) derives from the period prior to the advent of PAH-specific medical therapy and therefore may be inaccurate in estimating morbidity and mortality in the presence of newer medical therapies [10,11]. A more recent US demographic study, from the Registry to Evaluate Early and Long-Term PAH Disease Management (REVEAL) study group, found the mean age of onset of PAH to be 53 years old, with a higher female preponderance (79%) than prior registry data sets [4]. Concurrent with increased recognition of the disease, this changing demographic to an expanding at-risk older population (with higher rates of obesity and IR), suggests that the incidence of PAH could continue to escalate. Even with improvements in diagnosis, there is still a substantial delay (~2.8 years) between the time of symptom onset and subsequent invasive hemodynamic assessment [4]. This diagnostic delay may prove critical if there is delayed introduction of targeted therapies. With currently available medical treatment, survival rates at one year have increased from 68% to 91–97% [11,12]. Nonetheless, PAH remains a disease with poor long-term survival and no curative treatment or intervention is yet available.

**Figure 1:**
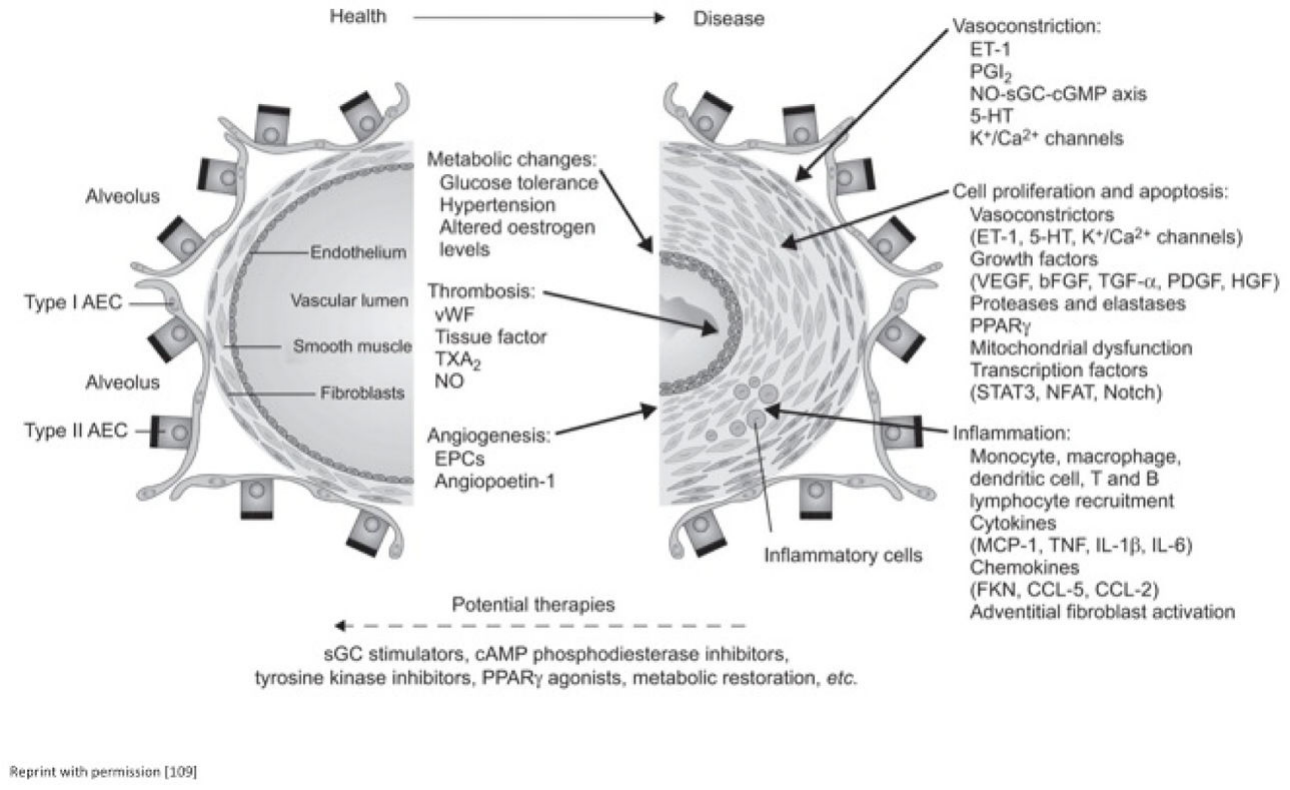
The key pathological mechanisms underlying vascular changes in pulmonary hypertension (PH). Potential new therapies for PH are also indicated. AEC: Alveolar Epithelial Cell; vWF: von Willebrand Factor; TXA2: Thromboxane A2; NO: Nitric oxide; EPC: Endothelial Progenitor Cell; ET-1: Endothelin-1; PGI2: Prostaglandin I2; sGC: Soluble Guanylate Cyclase; cGMP: Cyclic Guanosine Monophosphate; 5-HT: 5-hydroxytryptamine; VEGF: Vascular Endothelial Growth Factor; bFGF: Basic Fibroblast Growth Factor; TGF-a: Transforming Growth Factor-a; PDGF: Platelet-derived Growth Factor; HGF: Hepatocyte Growth Factor; PPARc: Peroxisome Proliferator-Activated Receptor-c;STAT3: Signal Transducer and Activator of Transcription 3; NFAT: Nuclear Factor of Activated T-cells; MCP-1: Monocyte Chemoattractant Protein-1; TNF: Tumour Necrosis Factor; IL: Interleukin; FKN: Fractalkine; CCL: Chemokine Ligand; cAMP: Cyclic Adenosine Monophosphate.

PAH is fundamentally a disorder of the pulmonary vasculature, but its pathogenesis involves the complex interplay of many different systems. PAH is a progressive disease characterized by aberrant endothelial function, changes in vascular tone, and disorganized vascular remodeling (secondary to the accumulation of immune and vascular cells within the lumen of arteries) (Figure 1). A complete understanding of the numerous factors behind these deleterious changes, however, is elusive. The classical view implicating an imbalance between vasodilatation and vasoconstriction appears to be overly simplistic to fully account for all of the changes that occur with PAH. Recent investigation into the cellular and molecular mechanisms of PAH have shed some light on the interplay between various vaso active factors, inflammatory mediators and growth inhibitors, and disordered platelet aggregation, all of which affect endothelial cells and vascular smooth muscle cells (SMCs) [13]. Numerous cytokines/chemokines, adipokines, growth factors, and transcriptional factors have been implicated (select factors in Table 2), reflecting the complicated multifactorial etiology driving the pathogenesis of PAH and providing a potential reason for differential presentation, etiology, and response to therapy [14]. Obesity and PAH appear to be related, although this association is not well understood.

## Obesity, Insulin Resistance and Inflammation

The prevalence of obesity has markedly increased during the last twenty years [15] and is associated with a wide-range of comorbidities affecting nearly every organ system. In spite of the well-acknowledged correlation between obesity and vascular disease, evidence linking obesity with acute and chronic pulmonary disease has only recently become evident [16]. Studies have also illustrated a relationship between obesity and PAH. Autopsies performed on overweight/obese subjects have revealed hypertensive changes in the pulmonary vasculature not present in normal weight controls [17]. In the REVEAL registry, the prevalence of idiopathic PAH was higher in overweight/obese subjects, a finding in dependent of the presence of other PAH-related conditions, such as sleep apnea, systemic hypertension and diastolic dysfunction [4]. Although far from conclusive, these findings suggest that obesity itself may contribute to the development and/or progression of PAH and could serve as an important marker of patient outcome.

Excess adiposity is an important cause of IR, one of major factors involved in the pathogenesis of Type 2 Diabetes Mellitus (T2DM) and a key component of the metabolic syndrome [18]. Although a large number of known and potentially unknown mechanisms underlie this relationship [19] recent discoveries have suggested that immune-mediated chronic inflammation contributes to the insulin-resistant state [20]. Obesity and related comorbidities (including cardiovascular and pulmonary disease) are associated with a state of chronic low-grade inflammation that can be detected both systemically and within specific tissues [21] and is now recognized as a major cause of decreased insulin sensitivity [22]. Inflammatory pathway activation has been observed in all classical insulin target tissues, including adipose, liver, skeletal muscle and the central nervous system, but also within the pulmonary vasculature and parenchyma [23–28].

In adipose tissue, macrophages play a central role in this phenomenon. Activation of a pro inflammatory pathway leads to the secretion of numerous cytokines, such as TNF-α, interleukin-6 (Il-6) and interleukin-1β (IL-1β) and down regulation of adiponectin [29,30]. These pro inflammatory cytokines not only induce changes in gene expression that affect metabolic regulation but also directly impair insulin signaling by binding to toll-like receptors (TLR2 and TLR4), ultimately leading to disruption in glucose uptake [31,32]. Pro inflammatory cytokines also impair suppression of adipose tissue lipolysis, leading to free fatty acid (FFA) release into the circulation [32–34], impaired insulin-stimulated muscle glucose uptake [35], and decreased suppression of hepatic glucose production [36]. One of the major cellular mechanism(s) responsible for FFA-induced IR involves activation of mammalian target of rapamycin (mTOR) [37], which inhibits protein kinase B (Akt), and ultimately prevents the translocation of GLUT-4 from the cytoplasm to the cell membrane for glucose transport [25,32,38–41]. Activation of the nuclear factor kappa B (NFκB) pathway, a major pro inflammatory pathway, has also been implicated in the pathogenesis of IR [25,42].

## Clinical Evidence of an Association Between PAH and IR

The first retrospective clinical report of a relationship between IR and PAH in adults was published in 2009. Using data from the National Health and Nutrition Examination Survey (NHANES), female participants with a diagnosis of PAH, irrespective of cause, were nearly twice as likely to be insulin resistant (determined by the triglyceride/high-density lipoprotein cholesterol (TG/HDL-C) ratio) compared to BMI-matched controls and had worse six month event-free survival [7]. In this analysis, the degree of TG/HDL-C elevation was independent of body mass index (BMI). This finding may simply reflect a poor correlation between lipoprotein concentration and IR, especially in certain minority ethnic groups [43], but may also indicate a dissociation between IR and obesity in the PAH population. A nearly simultaneous publication showed an increased incidence of the metabolic syndrome in patients with pulmonary venous hypertension (PVH), which was not unexpected given the comorbidities that are found with PVH, as evidenced by higher BMI, and increased rates of hypertension, hyperlipidemia, T2DM, and coronary artery disease, and does not necessarily imply a causal relationship [44]. A subsequent clinical report by the same authors looked at the prospective relationship between IR and PAH, rather than PVH [8]. In this cohort, glycated hemoglobin (HbA1c) was used to define glucose intolerance (6.0–6.4%) and T2DM (>6.5%). Over half (56%) of enrolled PAH patients had either impaired glucose tolerance or unrecognized T2DM. Despite this finding, there was no significant difference in six-month event-free survival based on the presence or absence of IR. From the same group, a recently published case report showed significant improvement in PAH after substantial weight loss induced by laparoscopic Roux-en-Y gastric bypass surgery [45]. In this case study, the subject had a 20% reduction in body fat mass over 20 months that was associated with a decrease in IR (2.6 to 1.2) as measured by the homeostasis model assessment of insulin resistance (HOMA-IR). This patient also experienced hemodynamic improvements in PAH, characterized by a reduction in PVR from approximately 6.5 to 3.5 Wood units, a decrease in mPA pressure from 60 to 35 mmHg, and improvement in New York Heart Association (NYHA) functional class and echocardiographic right heart findings. A similar case of another patient undergoing bariatric surgery, reported in 2008, also found hemodynamic improvements in PAH, but no measure of glucose tolerance was performed [46]. More robust dynamic measures of insulin sensitivity, such as the hyper insulinemic-euglycemic clamp procedure, have not been conducted in patients with PAH after weight loss, and may be useful in future research. Although bariatric surgery is not commonly performed in this population, due to the high surgical risk these patients pose, other weight loss interventions may be successful in improving the hemodynamic status of overweight and obese patients with PAH.

## Evidence Supporting the PAH-IR Association

**Figure 2:**
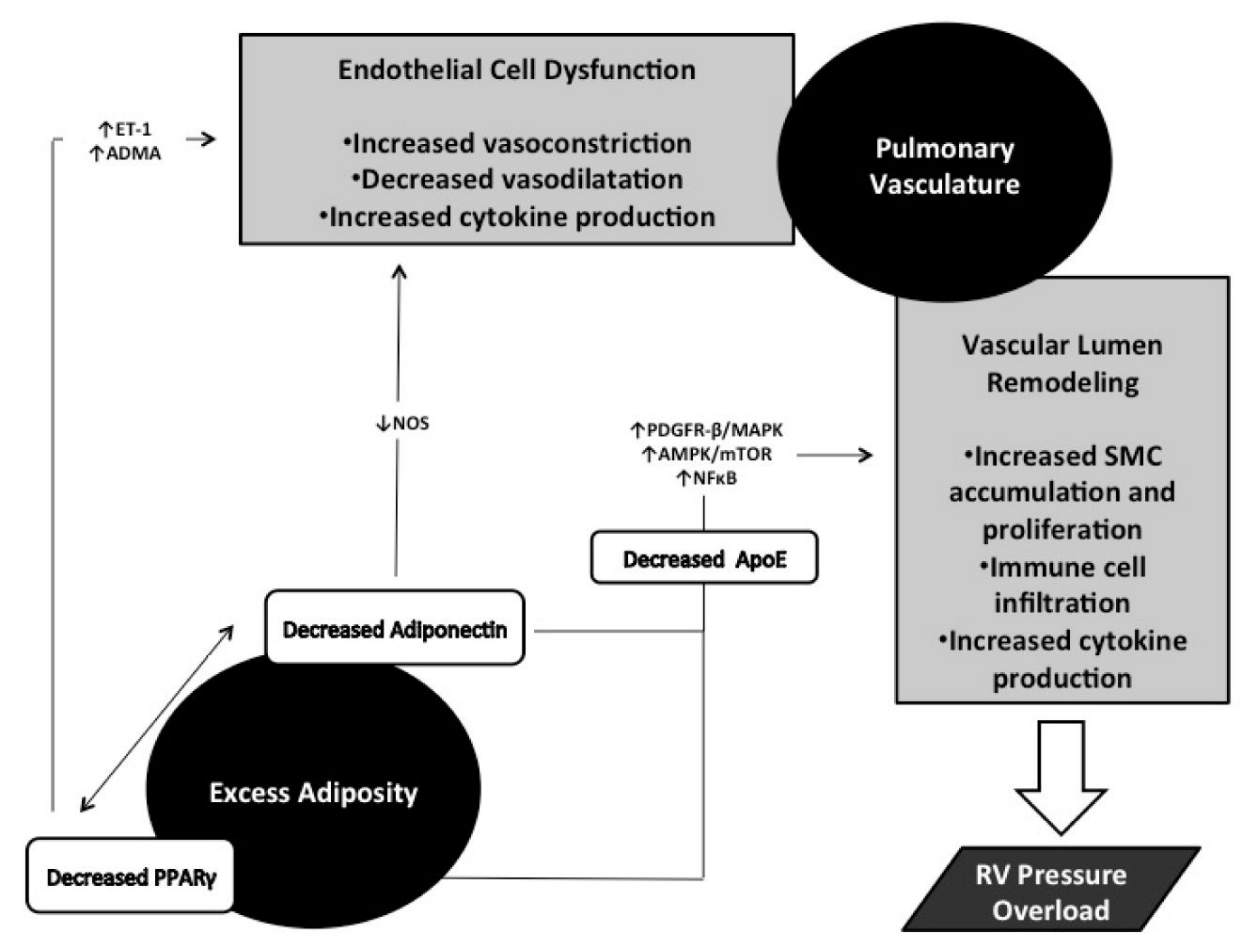
Potential pathways underlying the association between obesity-induced insulin resistance and pulmonary arterial hypertension. ET-1: Endothelin-1; ADMA: Asymmetric Dimethyl-Arginine; NOS: nNitric Oxide Synthase; PDGFR-β: Platelet-derived Growth Factor Receptor Beta; MAPK: Mitogen-activated Protein Kinase; AMPK: AMP Activated Protein Kinase; mTOR: Mammalian Target of Rapamycin; NFκB: Nuclear Factor Kappa-light-chain-Enhancer of Activated B cells; ApoE: Apolipoprotein E.

Alterations in several cytokines/chemokines and adipokines, the pathways that produce such factors, as well as local and distant co-factors which are present in both adipocytes and the pulmonary vasculature, have been implicated as potential mediators of PAH and its observed association with IR. The most extensively studied factors will be briefly reviewed here (Figure 2).

## Adiponectin

The importance of adipose tissue as an endocrine organ and dynamic mediator of metabolic processes is increasingly being recognized. Through the release of biologically active hormones (i.e. adipokines), adipocytes play a vital role in the development of both IR and obesity-associated chronic inflammation. One of the most important of these mediators, adiponectin, has key effects on metabolism, the immune system and the vasculature, all implicated in the pathogenesis of PAH.

Adiponectin is an almost exclusively adipocyte-derived peptide first characterized in 1996 and encoded by the ADIPOQ gene [47]. Circulating adiponectin exists predominantly in oligimeric form, but also as multiples of hexamers and trimers. The higher molecular weight oligomers appear to be more active in glycemic control [48]. Plasma levels of adiponectin are paradoxically reduced in obesity, the metabolic syndrome, T2DM and in the presence of CVD [49,50] and increased in response to weight loss and thiazolidinedione (TZD)-induced peroxisome proliferator-activated receptor-gamma (PPAR-γ) activation [51,52]. Mice deficient in adiponectin become insulin resistant in response to a high-fat diet and, in the presence of inflammatory cytokines, there is a notable decrease in adiponectin production [53–55]. In contrast, modestly increasing the levels of circulating adiponectin effectively reverses the diabetic phenotype in obese insulin-resistant ob/ob mice with reduced macrophage infiltration and systemic inflammation [56]. These, and other findings, point toward a prominent role for adiponectin in obesity-related inflammation and IR.

Adiponectin has important effects on many of the pathogenic processes underlying PAH development: increased vascular tone, vascular remodeling and angiogenesis, as well as chronic perivascular inflammation. Heightened vascular tone is a central finding in the progression of PAH, and available therapeutic agents all target this change through the process of vasodilatation or suppression of vasoconstriction. Adiponectin, independent of the presence of diabetes, has endothelium-dependent vasodilator properties, and conversely, a decrease in adiponectin levels leads to systemic hypertension [57]. Mice deficient in adiponectin have reduced levels of endothelial nitric oxide (one of the most potent vasodilators), a change also seen in experimental models of PAH [58]. Although direct evidence is lacking, these studies indicate a possible vasoactive role for adiponectinin PAH patients.

Platelet-derived growth factor (PDGF), epidermal growth factor (EGF) and vascular endothelial growth factor (VEGF) levels are increased in the pulmonary arteries of rodent models and PAH patients, leading to the subsequent production and accumulation of SMCs [59–61]. This SMC accumulation into the vascular lumen is critical to the development of both arterial muscularization/hypertrophy and plexi form lesion formation seen in PAH. Growth factor gene expression is increased in response to phosphorylation of platelet-derived growth factor receptor β (PDGFR-β) and the subsequent downstream activation of mitogen-activated protein kinases (MAPKs). Adiponectin inhibits PDGFR-β ligand binding, thus decreasing MAPK activation, and suppressing SMC migration and proliferation [62]. Evidence of this effect is apparent in rodent models of adiponectin deficiency, which exhibit increases in PDGF, VEGF, EGF and other mitogenic factors with SMC accumulation in the vascular lumen [59,61]. In addition to its direct effects on the pathway, adiponectin also alters the lung expression of the metabolically active PPAR-γ receptor [63] and affects circulating levels of ApoE [64], both of which are also involved in the PDGFR-β pathway (see below). Adiponectin also directly inhibits SMC proliferation by preventing growth-factor induced upregulation of the proinflammatory AMPK/mTOR pathway, common to both PAH and obesity-related inflammation and IR [65,66].

It is now well recognized that chronic inflammation contributes to the development of PAH. An increase in macrophage infiltration, a key component of the inflammatory response, is central to the development of hypoxia –induced PH in mice [67]. This infiltration is also seen in lung tissue of human subjects with idiopathic PAH [28]. Individuals with PAH have increased levels of circulating cytokines, which may help predict poor patient survival, along with enhanced migration and accumulation of other active immune cells, including T lymphocytes and mononuclear fibrocytes, into the pulmonary vasculature [27,28,68–71]. Adiponectin has potent anti-inflammatory effects in macrophages and in the vascular endothelium itself [72]. In the pulmonary vasculature and other tissues, adiponectin has also been shown to directly inhibit the proinflammatory mTOR and NFkB pathways and reduce peri vascular inflammatory cell infiltration [65,66,72–75].

Adiponectin has direct effects on glycemic control (decreased gluconeogenesis, increased peripheral glucose uptake) and lipid handling (decreased lipolysis, down regulation of lipogenesis, increased fatty acid beta (β)-oxidation) which serve to increase insulin sensitivity [76]. Levels of adiponectin, in particular the higher molecular weight forms, are inversely correlated with body fat percentage in adults [77,78]. Over expression of adiponectin in obese mice leads to improved glycemic control and adiponectin treatment, in combination with leptin, has been shown to reverse IR in mice [56,79]. Limited evidence indicates that certain metabolic abnormalities are more commonly seen in PAH patients, including IR and dyslipidemia which may contribute to poor patient outcomes [7].

Adiponectin deficiency is a common finding in both PAH and IR, perhaps providing a key link between the two disease states. This link may help explain, at least in part, the observed association between obesity and PAH and potentially lead to complementary and more effective treatments than are currently available.

## PPAR-γ and the BMP2/PDGFR/ApoE axis

PPAR-γ is a member of the nuclear-receptor super family that serves as a ligand-activated transcription factor for pancreatic β-cell, macrophage, and vascular endothelium gene expression. PPAR-γ is most highly expressed in adipose tissue, however, where it plays a prominent role in fatty acid storage and glucose metabolism [80]. Long-chain fatty acids and eicosanoids serve as endogenous ligands for the receptor, but it has also been well described as the target for the TZD class of medications that are useful in the treatment of T2DM and IR [81]. PPAR-γ ligand-activation initiates gene transcription either through transactivation or transrepression [80,82]. Transactivation is a DNA-dependent process whereby target PPAR response elements are bound into a heterodimeric complex with the retinoid X receptor (RXR), resulting in target gene transcription. Alternatively, transrepression is the DNA-independent process by which PPAR-γ is also activated, but this activation leads to repression of gene transcription. Through these two mechanisms, genes targeted by PPAR-γ encode many of the proteins implicated in the pathogenesis of PAH, such as adioponectin, endothelin – 1 (ET-1), monocyte chemotactic protein – 1 (MCP-1), interleukin – 6 (IL-6), and asymmetric dimethylarginine (ADMA, an endothelial nitric oxide synthase inhibitor), among others [63].

There is emerging evidence that PPAR-γ gene regulation plays an important role in the vascular remodeling and SMC proliferation seen in PAH. Central to this hypothesis is the finding that patients with PAH have reduced lung expression of PPAR-γ [83]. A major cause of heritable PAH is a genetic mutation in the bone morphogenetic protein receptor II (BMP-RII) [84]. This mutation leads to a decrease in PPAR-γ activity, an increase in MAPK activity, through the PDGFR-β pathway, and stimulation of vascular remodeling, one of the key findings in PAH. A decrease in PPAR-γ expression also causes dysregulation of endothelial cell cycle progression further contributing to enhanced vascular SMC proliferation [85,86]. In addition, PPAR-γ increases gene expression of adiponectin and ApoE, both of which further inhibit SMC proliferation by converging on the PDGFR-β pathway [63,64,87–89]. In this complex chain of events, adiponectin binds the PDGFR-β ligand, whereas ApoE internalizes PDGFR-β, with both processes serving to decrease the PDGFR-β ligand-mediated stimulation of SMC proliferation. This pathway, part of the BMP-RII/PPARγ/ApoE axis, may therefore be a key component in the observed PAH/IR association. ApoE, a protein with potent anti-atherogenic action has reduced pulmonary expression in PAH patients and experimental evidence indicates that ApoE-deficient mice fed a high fat diet exhibit features of insulin resistance [87,90,91].

As discussed previously, IR is associated with a chronic, low-grade inflammatory state that is evident in overweight/obese patients with PAH. This inflammatory state is accompanied by increased levels of cytokines/chemokines (IL-6, IL-1β, MCP-1 etc.) and decreased levels of adiponectin. These factors decrease insulin sensitivity through proinflammatory pathways such as NFκB. PPAR-γ acts to decrease NFκB signaling, T cell activation and impair production of harmful cytokines [92]. Many of these effects can be illustrated in studies involving the PPAR-γ agonist TZDs. These insulin-sensitizing agents have been shown to prevent vascular remodeling and atherosclerosis in the general systemic circulation [93–95]. Recent evidence, however, points to a direct role of TZDs in the pulmonary vasculature. Most notably, in a PAH rodent model, rosiglitazone reversed the changes observed in hypoxia-induced vascular remodeling [96].

PPAR-γ, in addition to adiponectin, may also have profound effects on the unopposed vasoconstriction that occurs with PAH. Pulmonary ET-1 expression and ADMA concentrations are increased in PAH subjects [98]. Cytokines secreted from perivascular adipocytes and macrophages inhibit nitric oxide production, a process which is predominantly mediated by ET-1 and to a smaller extent by ADMA. PPAR-γ acts to inhibit the gene expression of both ET-1 and ADMA, leading to vasodilatation [98].

## Limitations of Current Knowledge and Future Directions

PAH is a progressive disease with significant morbidity and mortality despite recent advances in diagnosis and treatment. Ultimately, PAH is a vascular disease, but many different organ systems and factors are involved in its pathogenesis. PAH is characterized by aberrant endothelial function, changes in vascular tone, and disorganized vascular remodeling. Recent investigation into the cellular and molecular mechanisms of PAH has highlighted the complex interaction between various vasoactive factors, growth inhibitors, and disorders of platelet aggregation that act upon target endothelial and vascular SMCs. In spite of these advances, it remains unlikely that one factor or pathway is solely responsible for all the changes seen in PAH. At present, there is limited understanding of the underlying biologic and environmental interactions that contribute to the disease and its varying clinical manifestations.

The association between obesity, IR and PAH deserves further investigation. Whether this simply represents an association, or a true cause-and-effect relationship, has yet to be determined and should be recognized as an important limitation to our current understanding. Pathways observed in obesity-associated IR are also active in the pulmonary vasculature of PAH patients. These recent findings open up the possibility that therapeutic interventions targeted to IR and obesity may one day prove useful in the treatment of PAH. Treatments based on the observed changes in adiponectin, ApoE and PPAR-γ, and their interrelated metabolic pathways, are promising. In addition, weight loss interventions (whether surgical, medical or lifestyle) could potentially improve, or even reverse, the pathophysiologic changes seen in overweight and obese PAH patients.

Isolated case reports have illustrated major hemodynamic improvements after substantial weight loss induced by bariatric surgery [45,46]. At this time, however, there is no direct evidence that treatment of obesity or insulin resistance changes the progression of disease or the response to PAH therapies. Animal studies suggest that PPAR-γ agonists can reverse the detrimental changes observed in the pulmonary vasculature, but no human studies are available at this time. In addition, heart failure is a relative contraindication to TZD use, and since most PAH patients have some degree of right ventricular systolic dysfunction (due to pressure overload from the pulmonary circulation), currently available TZD therapy is unlikely to be a viable treatment option for the majority of PAH patients. The role of other anti-diabetic medications also remains unclear. Incretin-based therapeutic agents (glucagon-like peptide-1 [GLP-1] agonists, Dipeptidyl peptidase-4 [DPP-4] inhibitors, etc.) have been shown to alter PPAR- γ expression in hepatic and adipose tissue and to mitigate IR, but their effects on the pulmonary vasculature in PAH subjects are unknown [99,100]. The biguanide drug, metformin, not only reduces endogenous glucose production from the liver and improves peripheral insulin sensitivity but also has an antiproliferative effect on cell cycle progression and may restore vascular reactivity [101–104]. In a rat model of PAH, metformin administration inhibited MAPK activation of SMC growth and proliferation but no human data is available and the potential for use in PAH patients is theoretical [105]. Limited animal data also supports a possible vasodilatory role for statin medications in PAH patients but definitive human data is again lacking [106,107].

In summary, data from basic science research, along with limited clinical evidence, supports a common pathophysiologic link between obesity, IR and PAH. Therapies targeting cytokine alterations, such as changes in PPAR-γ and adiponectin, appear promising but clinical studies on human subjects are scarce or nonexistent. Furthermore, the effect of these treatments on the specific molecular interactions involved in PAH is unpredictable and the ability to translate these interactions to changes in screening, treatment, and patient care is largely unknown and requires further research.

## Figures and Tables

**Table 1:** Updated Clinical Classification of Pulmonary Hypertension.

1 Pulmonary arterial hypertension (PAH)
1.1 Idiopathic PAH
1.2 Heritable
1.2.1 BMPR2
1.2.2 ALK1, endoglin (with or without hereditary hemorrhagic telangiectasia)
1.2.3 Unknown
1.3 Drug-and toxin-induced
1.4 Associated with
1.4.1 Connective tissue diseases
1.4.2 HIV infection
1.4.3 Portal hypertension
1.4.4 Congenital heart diseases
1.4.5 Schistosomiasis
1.4.6 Chronic hemolytic anemia
1.5 Persistent pulmonary hypertension of the newborn
1′ Pulmonary veno-occlusive disease (PVOD) and/or pulmonary capillary hemangiomatosis (PCH)
2 Pulmonary hypertension owing to left heart disease
2.1 Systolic dysfunction
2.2 Diastolic dysfunction
2.3 Valvular disease
3 Pulmonary hypertension owing to lung disease and/or hypoxia
3.1 Chronic obstructive pulmonary disease
3.2 Interstitial lung disease
3.3 Other pulmonary diseases with mixed restrictive and obstructive pattern
3.4 Sleep-disordered breathing
3.5 Alveolar hypoventilation disorders
3.6 Chronic exposure to high altitude
3.7 Developmental abnormalities
4 Chronic thromboembolic pulmonary hypertension (CTEPH)
5 Pulmonary hypertension with unclear multifactorial mechanisms
5.1 Hematologic disorders: myeloproliferative disorders, splenectomy
5.2 Systemic disorders: sarcoidosis, pulmonary Langerhans cell histiocytosis: lymphangioleiomyomatosis, neurofibromatosis, vasculitis
5.3 Metabolic disorders: glycogen storage disease, Gaucher disease, thyroid disorders
5.4 Others: tumoral obstruction, fibrosing mediastinitis, chronic renal failure on dialysis

Reprint with permission [5]

**Table 2:** Select factors implicated in the development of PAH.

Cytokine/Chemokine	Effect	Target effect	Location
Fractalkine (CX3CL1)	↑	Leukocyte recruitment	T cells
RANTES (CCL5)	↑	Attracts monocytes and T cells; induces ET - 1	EC
Endothelin converting enzyme – 1 (ET-1)	↑	Vasoconstriction and mitogenic action	EC
Monocyte chemotactic protein – 1 (MCP – 1, CCL2)	↑	Monocyte recruitment	EC, SMC
**Growth Factors**			
Platelet-derived growth factor (PDGF)	↑	Mitogen and chemoattractant for SMC, EC, fibroblasts; Resistance to apoptosis	EC
Epidermal growth factor (EGF)	↑	Induces proliferation and migration of SMC	EC, SMC, Macrophages
Vascular endothelial growth factor (VEGF)	↑	Induces proliferation and migration of SMC	EC
Seratonin (5-HT)	↑	Mitogenic effect on SMC, vasoconstriction	EC
Seratonin transporter (5-HTT)	↑	Co-mitogenic effect on SMC required for 5-HT action	SMC
Survivin	↑	Inhibitor of apoptosis	
**Transcriptional Factors**			
Nuclear factor of activated T cells (NFAT)	↑	Increases inflammatory mediators including several interleukins and TNFα, and inhibits apoptosis	T cells, SMC

EC: Endothelial cells, SMC: Smooth muscle cells, TNFα: Tumor necrosis factor α

## References

[1] WAGENVOORT CA (1960). Vasoconstriction and medial hypertrophy in pulmonary hypertension. Circulation.

[2] Palevsky HI, Schloo BL, Pietra GG, Weber KT, Janicki JS (1989). Primary pulmonary hypertension. Vascular structure, morphometry, and responsiveness to vasodilator agents. Circulation.

[3] Voelkel NF, Tuder RM, Weir EK (1997). Pathophysiology of primary pulmonary hypertension: from physiology to molecular mechanisms. Primary pulmonary hypertension.

[4] Badesch DB, Raskob GE, Elliott CG, Krichman AM, Farber HW (2010). Pulmonary arterial hypertension: baseline characteristics from the REVEAL Registry. Chest.

[5] Simonneau G, Robbins IM, Beghetti M, Channick RN, Delcroix M (2009). Updated clinical classification of pulmonary hypertension. J Am Coll Cardiol.

[6] McLaughlin VV, Archer SL, Badesch DB, Barst RJ, Farber HW (2009). ACCF/AHA 2009 expert consensus document on pulmonary hypertension a report of the American College of Cardiology Foundation Task Force on Expert Consensus Documents and the American Heart Association developed in collaboration with the American College of Chest Physicians; American Thoracic Society, Inc.; and the Pulmonary Hypertension Association. J Am Coll Cardiol.

[7] Zamanian RT, Hansmann G, Snook S, Lilienfeld D, Rappaport KM (2009). Insulin resistance in pulmonary arterial hypertension. Eur Respir J.

[8] Pugh ME, Robbins IM, Rice TW, West J, Newman JH (2011). Unrecognized glucose intolerance is common in pulmonary arterial hypertension. J Heart Lung Transplant.

[9] Humbert M, Sitbon O, Chaouat A, Bertocchi M, Habib G (2006). Pulmonary arterial hypertension in France: results from a national registry. Am J Respir Crit Care Med.

[10] Rich S, Dantzker DR, Ayres SM, Bergofsky EH, Brundage BH (1987). Primary pulmonary hypertension. A national prospective study. Ann Intern Med.

[11] D’Alonzo GE, Barst RJ, Ayres SM, Bergofsky EH, Brundage BH (1991). Survival in patients with primary pulmonary hypertension. Results from a national prospective registry. Ann Intern Med.

[12] Galiè N, Manes A, Uguccioni L, Serafini F, De Rosa M (1998). Primary pulmonary hypertension: insights into pathogenesis from epidemiology. Chest.

[13] Morrell NW, Adnot S, Archer SL, Dupuis J, Jones PL (2009). Cellular and molecular basis of pulmonary arterial hypertension. J Am Coll Cardiol.

[14] Hassoun PM, Mouthon L, Barberà JA, Eddahibi S, Flores SC (2009). Inflammation, growth factors, and pulmonary vascular remodeling. J Am Coll Cardiol.

[15] Ogden CL, Carroll MD, Curtin LR, McDowell MA, Tabak CJ (2006). Prevalence of overweight and obesity in the United States, 1999–2004. JAMA.

[16] Ware LB, Matthay MA (2000). The acute respiratory distress syndrome. N Engl J Med.

[17] Haque AK, Gadre S, Taylor J, Haque SA, Freeman D (2008). Pulmonary and cardiovascular complications of obesity: an autopsy study of 76 obese subjects. Arch Pathol Lab Med.

[18] DeFronzo RA (2004). Pathogenesis of type 2 diabetes mellitus. Med Clin North Am.

[19] Schenk S, Saberi M, Olefsky JM (2008). Insulin sensitivity: modulation by nutrients and inflammation. J Clin Invest.

[20] Winer S, Chan Y, Paltser G, Truong D, Tsui H (2009). Normalization of obesity-associated insulin resistance through immunotherapy. Nat Med.

[21] Romeo GR, Lee J, Shoelson SE (2012). Metabolic syndrome, insulin resistance, and roles of inflammation--mechanisms and therapeutic targets. Arterioscler Thromb Vasc Biol.

[22] Heilbronn LK, Campbell LV (2008). Adipose tissue macrophages, low grade inflammation and insulin resistance in human obesity. Curr Pharm Des.

[23] Weisberg SP, McCann D, Desai M, Rosenbaum M, Leibel RL (2003). Obesity is associated with macrophage accumulation in adipose tissue. J Clin Invest.

[24] Cai D, Yuan M, Frantz DF, Melendez PA, Hansen L (2005). Local and systemic insulin resistance resulting from hepatic activation of IKK-beta and NF-kappaB. Nat Med.

[25] Itani SI, Ruderman NB, Schmieder F, Boden G (2002). Lipid-induced insulin resistance in human muscle is associated with changes in diacylglycerol, protein kinase C, and IkappaB-alpha. Diabetes.

[26] Hanisch UK (2002). Microglia as a source and target of cytokines. Glia.

[27] Dorfmüller P, Humbert M, Perros F, Sanchez O, Simonneau G (2007). Fibrous remodeling of the pulmonary venous system in pulmonary arterial hypertension associated with connective tissue diseases. Hum Pathol.

[28] Tuder RM, Groves B, Badesch DB, Voelkel NF (1994). Exuberant endothelial cell growth and elements of inflammation are present in plexiform lesions of pulmonary hypertension. Am J Pathol.

[29] Olefsky JM, Glass CK (2010). Macrophages, inflammation, and insulin resistance. Annu Rev Physiol.

[30] Lazar MA (2006). The humoral side of insulin resistance. Nat Med.

[31] Osborn O, Olefsky JM (2012). The cellular and signaling networks linking the immune system and metabolism in disease. Nat Med.

[32] Holland WL, Bikman BT, Wang LP, Yuguang G, Sargent KM (2011). Lipid-induced insulin resistance mediated by the proinflammatory receptor TLR4 requires saturated fatty acid-induced ceramide biosynthesis in mice. J Clin Invest.

[33] Shulman GI (2000). Cellular mechanisms of insulin resistance. J Clin Invest.

[34] Boden G (2006). Fatty acid-induced inflammation and insulin resistance in skeletal muscle and liver. Curr Diab Rep.

[35] Kelley DE, Mokan M, Simoneau JA, Mandarino LJ (1993). Interaction between glucose and free fatty acid metabolism in human skeletal muscle. J Clin Invest.

[36] Ferrannini E, Barrett EJ, Bevilacqua S, DeFronzo RA (1983). Effect of fatty acids on glucose production and utilization in man. J Clin Invest.

[37] Um SH, D’Alessio D, Thomas G (2006). Nutrient overload, insulin resistance, and ribosomal protein S6 kinase 1, S6K1. Cell Metab.

[38] Boden G, Lebed B, Schatz M, Homko C, Lemieux S (2001). Effects of acute changes of plasma free fatty acids on intramyocellular fat content and insulin resistance in healthy subjects. Diabetes.

[39] Yu C, Chen Y, Cline GW, Zhang D, Zong H (2002). Mechanism by which fatty acids inhibit insulin activation of insulin receptor substrate-1 (IRS-1)-associated phosphatidylinositol 3-kinase activity in muscle. J Biol Chem.

[40] Holland WL, Knotts TA, Chavez JA, Wang LP, Hoehn KL (2007). Lipid mediators of insulin resistance. Nutr Rev.

[41] Wakelam MJ (1998). Diacylglycerol--when is it an intracellular messenger?. Biochim Biophys Acta.

[42] Barnes PJ, Karin M (1997). Nuclear factor-kappaB: a pivotal transcription factor in chronic inflammatory diseases. N Engl J Med.

[43] Giannini C, Santoro N, Caprio S, Kim G, Lartaud D (2011). The triglyceride-to-HDL cholesterol ratio: association with insulin resistance in obese youths of different ethnic backgrounds. Diabetes Care.

[44] Robbins IM, Newman JH, Johnson RF, Hemnes AR, Fremont RD (2009). Association of the metabolic syndrome with pulmonary venous hypertension. Chest.

[45] Pugh ME, Newman JH, Williams DB, Brittain E, Robbins IM (2013). Hemodynamic improvement of pulmonary arterial hypertension after bariatric surgery: potential role for metabolic regulation. Diabetes Care.

[46] Mathier MA, Zhang J, Ramanathan RC (2008). Dramatic functional improvement following bariatric surgery in a patient with pulmonary arterial hypertension and morbid obesity. Chest.

[47] Maeda K, Okubo K, Shimomura I, Funahashi T, Matsuzawa Y (1996). cDNA cloning and expression of a novel adipose specific collagen-like factor, apM1 (AdiPose Most abundant Gene transcript 1). Biochem Biophys Res Commun.

[48] Waki H, Yamauchi T, Kamon J, Ito Y, Uchida S (2003). Impaired multimerization of human adiponectin mutants associated with diabetes. Molecular structure and multimer formation of adiponectin. J Biol Chem.

[49] Kumada M, Kihara S, Sumitsuji S, Kawamoto T, Matsumoto S (2003). Association of hypoadiponectinemia with coronary artery disease in men. Arterioscler Thromb Vasc Biol.

[50] Oh DK, Ciaraldi T, Henry RR (2007). Adiponectin in health and disease. Diabetes Obes Metab.

[51] Hotta K, Funahashi T, Arita Y, Takahashi M, Matsuda M (2000). Plasma concentrations of a novel, adipose-specific protein, adiponectin, in type 2 diabetic patients. Arterioscler Thromb Vasc Biol.

[52] Maeda N, Takahashi M, Funahashi T, Kihara S, Nishizawa H (2001). PPARgamma ligands increase expression and plasma concentrations of adiponectin, an adipose-derived protein. Diabetes.

[53] Maeda N, Shimomura I, Kishida K, Nishizawa H, Matsuda M (2002). Diet-induced insulin resistance in mice lacking adiponectin/ACRP30. Nat Med.

[54] Nawrocki AR, Rajala MW, Tomas E, Pajvani UB, Saha AK (2006). Mice lacking adiponectin show decreased hepatic insulin sensitivity and reduced responsiveness to peroxisome proliferator-activated receptor gamma agonists. J Biol Chem.

[55] Takemura Y, Walsh K, Ouchi N (2007). Adiponectin and cardiovascular inflammatory responses. Curr Atheroscler Rep.

[56] Kim JY, van de Wall E, Laplante M, Azzara A, Trujillo ME (2007). Obesity-associated improvements in metabolic profile through expansion of adipose tissue. J Clin Invest.

[57] Tan KC, Xu A, Chow WS, Lam MC, Ai VH (2004). Hypoadiponectinemia is associated with impaired endothelium-dependent vasodilation. J Clin Endocrinol Metab.

[58] Ouedraogo R, Gong Y, Berzins B, Wu X, Mahadev K (2007). Adiponectin deficiency increases leukocyte-endothelium interactions via upregulation of endothelial cell adhesion molecules in vivo. J Clin Invest.

[59] Partovian C, Adnot S, Raffestin B, Louzier V, Levame M (2000). Adenovirus-mediated lung vascular endothelial growth factor overexpression protects against hypoxic pulmonary hypertension in rats. Am J Respir Cell Mol Biol.

[60] Perros F, Montani D, Dorfmüller P, Durand-Gasselin I, Tcherakian C (2008). Platelet-derived growth factor expression and function in idiopathic pulmonary arterial hypertension. Am J Respir Crit Care Med.

[61] Schermuly RT, Dony E, Ghofrani HA, Pullamsetti S, Savai R (2005). Reversal of experimental pulmonary hypertension by PDGF inhibition. J Clin Invest.

[62] Arita Y, Kihara S, Ouchi N, Maeda K, Kuriyama H (2002). Adipocyte-derived plasma protein adioonectin acts as a platelet-derived growth factor-BB-binding protein and regulates growth factor-induced common postreceptor signal in vascular smooth muscle cell. Circulation.

[63] Hansmann G, Zamanian RT (2009). PPARgamma activation: a potential treatment for pulmonary hypertension. Sci Transl Med.

[64] Hansmann G, Wagner RA, Schellong S, Perez VA, Urashima T (2007). Pulmonary arterial hypertension is linked to insulin resistance and reversed by peroxisome proliferator-activated receptor-gamma activation. Circulation.

[65] Ding M, Xie Y, Wagner RJ, Jin Y, Carrao AC (2011). Adiponectin induces vascular smooth muscle cell differentiation via repression of mammalian target of rapamycin complex 1 and FoxO4. Arterioscler Thromb Vasc Biol.

[66] Wang C, Mao X, Wang L, Liu M, Wetzel MD (2007). Adiponectin sensitizes insulin signaling by reducing p70 S6 kinase-mediated serine phosphorylation of IRS-1. J Biol Chem.

[67] Vergadi E, Chang MS, Lee C, Liang OD, Liu X (2011). Early macrophage recruitment and alternative activation are critical for the later development of hypoxia-induced pulmonary hypertension. Circulation.

[68] Humbert M, Monti G, Brenot F, Sitbon O, Portier A (1995). Increased interleukin-1 and interleukin-6 serum concentrations in severe primary pulmonary hypertension. Am J Respir Crit Care Med.

[69] Fartoukh M, Emilie D, Le Gall C, Monti G, Simonneau G (1998). Chemokine macrophage inflammatory protein-1alpha mRNA expression in lung biopsy specimens of primary pulmonary hypertension. Chest.

[70] Soon E, Holmes AM, Treacy CM, Doughty NJ, Southgate L (2010). Elevated levels of inflammatory cytokines predict survival in idiopathic and familial pulmonary arterial hypertension. Circulation.

[71] Nicolls MR, Taraseviciene-Stewart L, Rai PR, Badesch DB, Voelkel NF (2005). Autoimmunity and pulmonary hypertension: a perspective. Eur Respir J.

[72] Summer R, Fiack CA, Ikeda Y, Sato K, Dwyer D (2009). Adiponectin deficiency: a model of pulmonary hypertension associated with pulmonary vascular disease. Am J Physiol Lung Cell Mol Physiol.

[73] Sugiyama M, Takahashi H, Hosono K, Endo H, Kato S (2009). Adiponectin inhibits colorectal cancer cell growth through the AMPK/mTOR pathway. Int J Oncol.

[74] Wu L, Xu B, Fan W, Zhu X, Wang G (2013). Adiponectin protects Leydig cells against proinflammatory cytokines by suppressing the nuclear factor-κB signaling pathway. FEBS J.

[75] Medoff BD, Okamoto Y, Leyton P, Weng M, Sandall BP (2009). Adiponectin deficiency increases allergic airway inflammation and pulmonary vascular remodeling. Am J Respir Cell Mol Biol.

[76] Ye R, Scherer PE (2013). Adiponectin, driver or passenger on the road to insulin sensitivity?. Mol Metab.

[77] Ukkola O, Santaniemi M (2002). Adiponectin: a link between excess adiposity and associated comorbidities?. J Mol Med (Berl).

[78] Nedvídková J, Smitka K, Kopský V, Hainer V (2005). Adiponectin, an adipocyte-derived protein. Physiol Res.

[79] Yamauchi T, Kamon J, Waki H, Terauchi Y, Kubota N (2001). The fat-derived hormone adiponectin reverses insulin resistance associated with both lipoatrophy and obesity. Nat Med.

[80] Willson TM, Lambert MH, Kliewer SA (2001). Peroxisome proliferator-activated receptor gamma and metabolic disease. Annu Rev Biochem.

[81] Yki-Järvinen H (2004). Thiazolidinediones. N Engl J Med.

[82] Chinetti G, Fruchart JC, Staels B (2000). Peroxisome proliferator-activated receptors (PPARs): nuclear receptors at the crossroads between lipid metabolism and inflammation. Inflamm Res.

[83] Ameshima S, Golpon H, Cool CD, Chan D, Vandivier RW (2003). Peroxisome proliferator-activated receptor gamma (PPARgamma) expression is decreased in pulmonary hypertension and affects endothelial cell growth. Circ Res.

[84] Machado RD, Eickelberg O, Elliott CG, Geraci MW, Hanaoka M (2009). Genetics and genomics of pulmonary arterial hypertension. J Am Coll Cardiol.

[85] Wakino S, Kintscher U, Kim S, Yin F, Hsueh WA (2000). Peroxisome proliferator-activated receptor gamma ligands in hibit retinoblastoma phosphyorylation and G1-->S transition in vascular smooth muscle cells. J Biol Chem.

[86] Ishigami M, Swertfeger DK, Granholm NA, Hui DY (1998). Apolipoprotein E inhibits platelet-derived growth factor-induced vascular smooth muscle cell migration and proliferation by suppressing signal transduction and preventing cell entry to G1 phase. J Biol Chem.

[87] Hansmann G, de Jesus Perez VA, Alastalo TP, Alvira CM, Guignabert C (2008). An antiproliferative BMP-2/PPARgamma/apoE axis in human and murine SMCs and its role in pulmonary hypertension. J Clin Invest.

[88] Sharabi Y, Oron-Herman M, Kamari Y, Avni I, Peleg E (2007). Effect of PPAR-gamma agonist on adiponectin levels in the metabolic syndrome: lessons from the high fructose fed rat model. Am J Hypertens.

[89] Ouchi N, Kihara S, Funahashi T, Matsuzawa Y, Walsh K (2003). Obesity, adiponectin and vascular inflammatory disease. Curr Opin Lipidol.

[90] Greenow K, Pearce NJ, Ramji DP (2005). The key role of apolipoprotein E in atherosclerosis. J Mol Med (Berl).

[91] Geraci MW, Moore M, Gesell T, Yeager ME, Alger L (2001). Gene expression patterns in the lungs of patients with primary pulmonary hypertension: a gene microarray analysis. Circ Res.

[92] Duan SZ, Usher MG, Mortensen RM (2008). Peroxisome proliferator-activated receptor-gamma-mediated effects in the vasculature. Circ Res.

[93] Buchanan TA, Meehan WP, Jeng YY, Yang D, Chan TM (1995). Blood pressure lowering by pioglitazone. Evidence for a direct vascular effect. J Clin Invest.

[94] Law RE, Meehan WP, Xi XP, Graf K, Wuthrich DA (1996). Troglitazone inhibits vascular smooth muscle cell growth and intimal hyperplasia. J Clin Invest.

[95] Chen Z, Ishibashi S, Perrey S, Osuga Ji, Gotoda T (2001). Troglitazone inhibits atherosclerosis in apolipoprotein E-knockout mice: pleiotropic effects on CD36 expression and HDL. Arterioscler Thromb Vasc Biol.

[96] Crossno JT, Garat CV, Reusch JE, Morris KG, Dempsey EC (2007). Rosiglitazone attenuates hypoxia-induced pulmonary arterial remodeling. Am J Physiol Lung Cell Mol Physiol.

[97] Kielstein JT, Bode-Böger SM, Hesse G, Martens-Lobenhoffer J, Takacs A (2005). Asymmetrical dimethylarginine in idiopathic pulmonary arterial hypertension. Arterioscler Thromb Vasc Biol.

[98] Yudkin JS, Eringa E, Stehouwer CD (2005). “Vasocrine” signalling from perivascular fat: a mechanism linking insulin resistance to vascular disease. Lancet.

[99] Svegliati-Baroni G, Saccomanno S, Rychlicki C, Agostinelli L, De Minicis S (2011). Glucagon-like peptide-1 receptor activation stimulates hepatic lipid oxidation and restores hepatic signaling alteration induced by a high-fat diet in nonalcoholic steatohepatitis. Liver International Liver Int.

[100] Shimasaki T, Masaki T, Mitsutomi K, Ueno D, Gotoh K (2013). The dipeptidyl peptidase-4 inhibitor des-fluoro-sitagliptin regulates brown adipose tissue uncoupling protein levels in mice with diet-induced obesity. PLoS One.

[101] Caspary WF, Creutzfeldt W (1971). Analysis of the inhibitory effect of biguanides on glucose absorption: inhibition of active sugar transport. Diabetologia.

[102] Borst SE, Snellen HG (2001). Metformin, but not exercise training, increases insulin responsiveness in skeletal muscle of Sprague-Dawley rats. Life Sci.

[103] Ben Sahra I, Laurent K, Loubat A, Giorgetti-Peraldi S, Colosetti P (2008). The antidiabetic drug metformin exerts an antitumoral effect in vitro and in vivo through a decrease of cyclin D1 level. Oncogene.

[104] Sartoretto JL, Melo GA, Carvalho MH, Nigro D, Passaglia RT (2005). Metformin treatment restores the altered microvascular reactivity in neonatal streptozotocin-induced diabetic rats increasing NOS activity, but not NOS expression. Life Sci.

[105] Agard C, Rolli-Derkinderen M, Dumas-de-La-Roque E, Rio M, Sagan C (2009). Protective role of the antidiabetic drug metformin against chronic experimental pulmonary hypertension. Br J Pharmacol.

[106] Li XL, Guan RJ, Li JJ (2012). Attenuation of monocrotaline-induced pulmonary arterial hypertension in rats by rosuvastatin. J Cardiovasc Pharmacol.

[107] Schermuly RT, Ghofrani HA, Wilkins MR, Grimminger F (2011). Mechanisms of disease: pulmonary arterial hypertension. Nat Rev Cardiol.

